# Effects of Power-Oriented Resistance Training During an Altitude Camp on Strength and Technical Performance of Elite Judokas

**DOI:** 10.3389/fphys.2021.606191

**Published:** 2021-02-18

**Authors:** Filipa Almeida, Paulino Padial, Juan Bonitch-Góngora, Blanca de la Fuente, Brad J. Schoenfeld, Antonio J. Morales-Artacho, Cristina Benavente, Belén Feriche

**Affiliations:** ^1^Department of Physical Education and Sport, University of Granada, Granada, Spain; ^2^High Performance Center of Sierra Nevada, Spanish Sport Council, Granada, Spain; ^3^Department of Health Sciences, CUNY Lehman College, New York, NY, United States; ^4^Laboratory Sport, Expertise and Performance (EA 7370), Research Department, French Institute of Sport (INSEP), Paris, France

**Keywords:** judo, hypoxia, altitude training camp, technique, muscle power

## Abstract

This study investigated the effect of a 3-week power-oriented resistance training program performed at moderate altitude on leg power output variables in a countermovement jump, a related judo technique (*ippon-seoi-nage*) and the relationship between them. Twenty-four elite male judokas were randomly assigned to a hypobaric hypoxia or normoxia group. Mechanical outputs from an incremental loaded countermovement jump test and the kinematic variables transferred to a dummy during an *ippon-seoi-nage* test (time to execution and movement accelerations) were assessed before, after, 1 and 2 weeks after training. Results indicated an increase in explosive leg capacity both at moderate altitude (2320 m.a.s.l.) and sea level. The hypoxia group showed additional benefits when compared to normoxia group for peak velocities with different percentages of the body weight, maximal theoretical velocity and jump height after the training period, and these additional benefits in jump height were maintained 2 weeks after training. The hypoxia group achieved a higher peak performance in peak velocity and jump height than normoxia group (peak velocity: 8.8 vs. 5.6%, jump height: 8.2 vs. 1.4%, respectively) and was achieved earlier in hypoxia (after training) than in normoxia (1 week after training). However, there was a detrimental effect for the hypoxia group on the times of execution and acceleration of the *ippon-seoi-nage* compared to the normoxia group. These results suggest that altitude training may induce faster and greater improvements in explosive leg extension capacity. Specific technique-oriented training should be included at altitude to prevent technique impairment.

## Introduction

**FIGURE 1:**
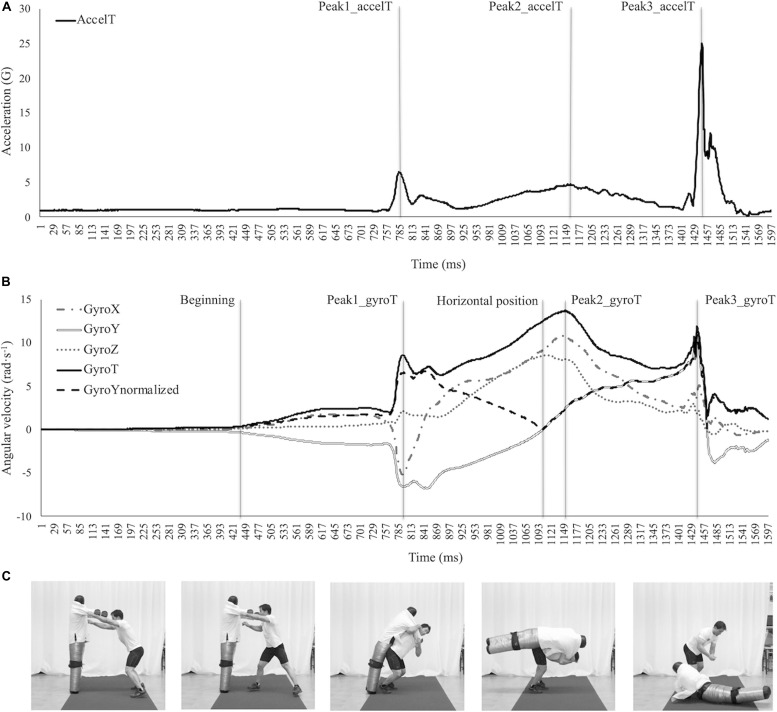
Representation of the resultant acceleration (AccelT) **(A)**, angular velocity in the three axes (GyroX, GyroY, and GyroZ) and resultant angular velocity (GyroT) **(B)** linked to the sequence of the *ippon-seoi-nage* performed by one judoka **(C)**. Three landmarks of the AccelT (Peak1_accelT, Peak2_accelT, and Peak3_accelT) and of the GyroT (Peak1_gyroT, Peak2_gyroT, and Peak3_gyroT) are displayed. The beginning of the repetition (considering the baseline of the GyroY) and the dummy’s horizontal position (represented by the inflection point on the GyroY normalized) are also displayed (Almeida et al., 2018).

Strength and power levels are widely considered a potential predictor of judo performance. Moreover, countermovement jump height values can be used to differentiate judokas of different training levels (Detanico et al., 2016). Throwing techniques are known to require high levels of strength and power to be performed at high velocities and against a great resistance from the opponent (Bonitch-Domínguez et al., 2010; Franchini et al., 2011). To perform *ippon-seoi-nage*, which is one of the most commonly used techniques in judo (Ishii et al., 2018), the judoka has to pull the *judogi* (judo suit) of the *uke* (person who is thrown) to render him off-balance, turning to position himself underneath the *uke* before finally throwing him over his shoulder (Figure 1C). The leg extension action has been considered of paramount importance in the technical performance of *ippon-seoi-nage* (Ishii et al., 2018), being responsible for the last part of this technique. It is important to note that the leg extension phase of the *ippon-seoi-nage* (Almeida et al., 2018) may be enhanced by improvements in explosive leg extension capacity (i.e., capacity to explosively extend the knee and hip). Therefore, the application of a power-oriented training program that enhances leg pushing capacity of judokas is crucial to improve performance.

The potential influence of environmental hypoxia on acute and chronic responses to strength training and on post-altitude muscle behavior remains largely unexplored. Exposure to higher altitudes generally has been regarded as counterproductive to muscle development (Ferretti et al., 1990; Narici and Kayser, 1995). However, research in the last decade has challenged this dogma (Scott et al., 2014a, 2017; Feriche et al., 2017), suggesting that exposure to moderate hypoxia may help to maximize muscle strength adaptations. Emerging evidence supports the use of moderate altitude training to improve leg extension power capacity at sea level (García-Ramos et al., 2014, 2016a,b; Feriche et al., 2017), whilst doubts remain as to the principal mechanisms involved (Millet et al., 2012; Feriche et al., 2017). Moreover, altitude training is expected to improve both physical and technical capacity of judokas, since the reduction in air resistance (Levine et al., 2008; Hamlin et al., 2015), the enhanced motor unit recruitment due to a higher reliance on anaerobic metabolism (Billaut et al., 2012; Schoenfeld, 2013) and the increase in spinal excitability (Delliaux and Jammes, 2006; Tomazin et al., 2016) have been linked to improvements in explosive actions.

To date, no research has investigated the effects of altitude training in judokas, or the duration of altitude training effects on these athletes after returning to sea level. Therefore, the aim of this study was to analyze the effect of a lower-limb power-oriented training program at moderate altitude on the kinematic variables transferred to a dummy during the *ippon-seoi-nage* and on the mechanical outputs of a countermovement jump. We hypothesized that: (1) altitude training would improve countermovement jump output; and (2) both the altitude training effect on countermovement jump output and on the kinematic variables of the *ippon-seoi-nage* would improve performance of this technique.

## Materials and Methods

### Participants

Twenty-nine participants initially agreed to participate in the study. Subsequently, five participants withdrew due to injury or inability to complete the training program, mainly as a result of unexpected judo competitions that occurred during the course of the intervention. Thus, 24 male judokas from the Spanish Judo Training Center of Valencia (age: 22.04 ± 3.18 years; body mass: 84.54 ± 19.17 kg; height: 179.36 ± 9.84 cm; fat percentage: 11.83 ± 3.28%) participated in this study. The study was carried out at the end of a special preparation mesocycle, in which the main aim was to improve muscle power (Cormie et al., 2011) so as to enhance the specific performance of the judokas before competition (Bonitch-Domínguez et al., 2010). All participants had experience in the loaded countermovement jump and in the protocol used in this study. They had been practicing judo for at least 10 years and they all had achieved the level of black belt (from first to third Dan). All of them have been medalists in junior or senior National Championships in Spain, Dominican Republic or Georgia; eight of them in junior or senior European Cups; four in Continental Opens; one in Grand Prix; two in junior Continental Championships; and one in junior World Championships. They reported no chronic diseases or recent injuries that could compromise performance. Participants did not have previous altitude training experience and they had not been exposed to altitudes above 1500 m.a.s.l. for more than 3–4 consecutive days for at least 2 months before the study. Participants were instructed to avoid any strenuous exercise for a minimum of 2 days preceding the testing sessions. They also were advised to maintain their customary nutritional intake and to avoid consuming any potentially ergogenic supplements during the course of the study. All participants were informed about the study protocol and signed a written informed consent form prior to investigation. The study protocol was approved by the university Institutional Review Board and was in accordance with the Declaration of Helsinki.

### Experimental Design

We used a longitudinal design, with intra and inter-group measurements, to compare the effect of a lower-limb power-oriented resistance training at moderate natural altitude (hypobaric hypoxia) or sea level (normoxia) on explosive leg extension capacity during a countermovement jump and kinematic variables of a related judo technique (*ippon-seoi-nage*) in elite judokas. Participants were randomly assigned to a group that performed a 3-week training program at hypobaric hypoxia (at the High Performance Center of Sierra Nevada, 2320 m.a.s.l.; hypoxia group; *n* = 13) or normoxia (at the Spanish Judo Training Center of Valencia, 15 m.a.s.l.; normoxia group; *n* = 11). Testing sessions were conducted under normoxic conditions at four time points: pre-training (Pre), post-training (Post-0), one (Post-1), and 2 weeks (Post-2) after training. All tests were performed in the same order (countermovement jump test and then *ippon-seoi-nage* test), at the same time of day and at similar environmental conditions.

### Procedures

#### Countermovement Jump Test

After a 10-min standardized warm-up (jogging, dynamic stretching, joint mobility exercises, unloaded countermovement jumps, and five countermovement jumps loaded with 20 kg), participants undertook an incremental loaded countermovement jump test. The protocol consisted of two repetitions per each loading condition (20, 40, 60, and 80 kg), separated by 1 min of rest between repetitions with the same load and 3 min between different loading conditions. A complete description of the countermovement jump technique can be found elsewhere (Almeida et al., 2018). The test was performed in a Smith machine (Multipower Fitness Line, Peroga, Murcia, Spain) with a linear velocity transducer (T-Force System, Ergotech, Murcia, Spain) using a 1000 Hz sampling rate attached to the bar. The peak velocity (the highest instantaneous velocity registered during the jump) and the mean propulsive velocity [the mean velocity registered during the propulsive phase of the movement, defined as the portion of the concentric phase during which the measured acceleration is greater than the acceleration due to gravity (acceleration ≥ −9.81 m⋅ s^–2^)] of each jump were recorded. The repetition with the highest peak velocity of each load was selected and used for analysis. The relationship between load displaced and peak velocity was established by fitting first-order-polynomials to the data. Peak velocity related to the load displacement equivalent to the 25, 50, 75, and 100% of the judoka’s body mass was calculated through a regression equation. The mean propulsive velocity and load values from each jump were used to assess another individual load-velocity relationship through a linear regression. Subsequently, the 1RM was considered as the load linked to a mean propulsive velocity of 0.33 m.s^–1^ (Conceição et al., 2016; Loturco et al., 2016). The 1RM in normalized values (kg.kg^–1^ of body mass), the maximal theoretical velocity (V_0_, velocity-axis intercept), the maximal theoretical load (L_0_, load-axis intercept), the load-velocity relationship slope (slope = −L_0_/V_0_) were subsequently determined. Additionally, two repetitions of a free countermovement jump with a plastic bar (0.2 kg) were performed before the beginning of the loaded incremental countermovement jump test. The jump height was estimated from the flight time collected by an infrared platform (Optojump, Microgate, Bolzano, Italy) at a 1000 Hz sampling rate. The highest jump was selected and used for analysis.

#### *Ippon-seoi-nage* Test

Participants performed a specific warm-up (five *ippon-seoi-nage* repetitions) to prepare for the technique test. During the warm-up and the technique test, participants used a dummy as an *uke* (57 kg of mass) to ensure stable execution conditions during all the assessments. The technique test began after 3 min of rest and included three repetitions of the *ippon-seoi-nage* with 1 min of rest between attempts. A complete description of the *ippon-seoi-nage* is provided elsewhere (Almeida et al., 2018). Kinematic variables transferred to the *uke* during the *ippon-seoi-nage* technique test were assessed by using a wearable sensor (Wimu, Realtrack System, Almería, Spain) placed on the back of the dummy. The sensor was fixed with a belt at waist height. This placement was considered as the center of mass and ensured that the sensor was protected from direct impact from the judoka or the floor. The device analyzed acceleration (G) and angular velocity (rad⋅s^–1^) in the three axes (*x* or longitudinal axis, *y* or transversal axis, and *z* or anterior-posterior axis) at a 1000 Hz sampling rate (Figure 1). The beginning of the repetition was defined as the time when the dummy started to become unbalanced (i.e., the angular velocity in the *y*-axis deviates from the baseline with a permanent change of at least 50 ms). Three peaks of the resultant acceleration (AccelT) were determined, the first related to the off-balance (Peak1_accelT), the second to the judoka’s leg extension (Peak2_accelT) and the third to the dummy’s impact on the ground (Peak3_accelT) (Figure 1A). The studied variables were organized as (1) time variables (T): time to reach the first, second and third peak of the resultant acceleration (Tpeak1_accelT, Tpeak2_accelT, and Tpeak3_accelT, respectively), and time to reach the dummy’s horizontal position (Thor); and (2) acceleration variables: values of resultant acceleration in the first (Max1_accelT), second (Max2_accelT), and third (Max3_accelT) peaks. A video camera, Casio EX-F1 (Tokyo, Japan), was used to record the technical testing sessions at a 250 Hz sampling rate. Two experienced coaches rated the three *ippon-seoi-nage* repetitions based on the technical model approach of the Kodokan School (Daigo, 2005), displaying a very high interrater reliability (Cronbach’s alpha = 0.991). The worst repetition was excluded and the repetition with the quickest time to reach the dummy’s horizontal position was chosen as the best repetition for analysis.

#### Training Program

**FIGURE 2:**
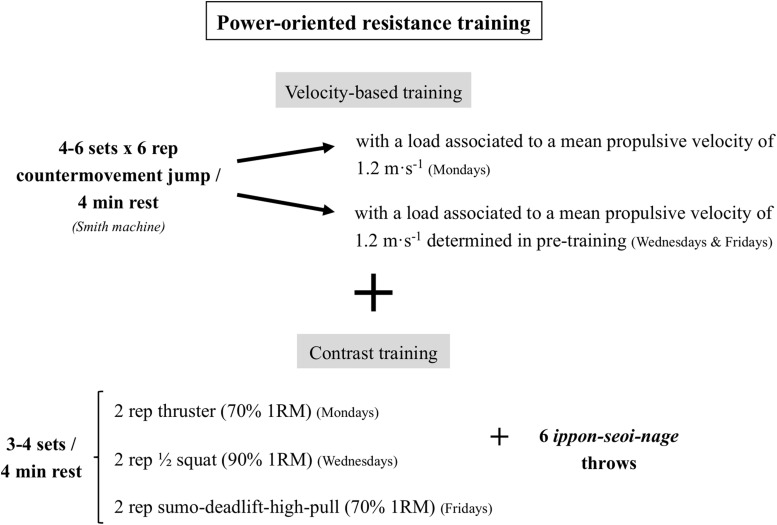
Power-oriented resistance training program.

The 3-week training program included a physical conditioning session in the morning and a judo session in the afternoon, from Monday to Saturday morning. Time and perceived exertion (RPE) were monitored in all sessions. Within 30 min after each session, a category RPE scale ranging from 0 to 10 (Foster et al., 2001) was used to assess training intensity. Participants performed a total of 14 judo sessions during the training camp. Those sessions were agreed upon by the coaches, maintaining the same exercises and volume. The judo training intensity was slightly reduced in the hypoxia group in the first week to favor the adaptation to altitude training (RPE of 5.83 ± 0.70 in hypoxia group vs. 6.59 ± 0.55 in normoxia group; *p* = 0.01). This intensity was progressively increased reaching the same intensity as normoxia group in the third week (RPE of 6.05 ± 0.97 in hypoxia group vs. 6.56 ± 0.45 in normoxia group; *p* = 0.10). The physical conditioning training included a total of eight power-oriented resistance sessions alternated with seven metabolic sessions. The metabolic sessions comprised a high-intensity circuit-training routine of short, medium, or long duration (10–20 min), as well as compensatory exercises. The content of the physical conditioning training was designed and supervised by the research team. Figure 2 shows the content of the power-oriented resistance sessions. The training load displaced during all countermovement jumps (∼35–40% 1RM) was estimated from the load linked to 1.2 m⋅s^–1^ of the mean propulsive velocity from the individual load-velocity relationship (Pérez-Castilla et al., 2020). For this, a linear regression model from a three-load incremental test was fitted each Monday, after the warm-up, and used to estimate the new weekly external load corresponding to a barbell mean propulsive velocity of 1.2 m⋅s^–1^. Wednesdays and Fridays’ training load was estimated during pre-training (Pre), which allowed the participants to improve the velocity as their explosive leg extension capacity increased in both study conditions. Both groups showed similar RPE values in the resistance (5.51 ± 0.89 in hypoxia group vs. 5.46 ± 0.66 in normoxia group; *p* = 0.86) and metabolic sessions (6.67 ± 0.65 in hypoxia group vs. 6.65 ± 0.47 in normoxia group; *p* = 0.95). The mean duration of the sessions was similar between groups and was 105.01 ± 16.58 min for the judo sessions and 78.29 ± 4.45 min for the physical conditioning sessions. After the training camp there were two training-controlled weeks. During this period, both groups trained together in the Spanish Judo Training Center of Valencia, completing 10 judo sessions and 9 physical conditioning sessions (seven resistance and two metabolic sessions). The mean duration of these sessions was 114.92 ± 9.44 min for the judo sessions and 78.56 ± 2.87 min for the physical conditioning sessions. Similar RPE values were found during the post-training camp period between groups in the judo (6.88 ± 0.83 in hypoxia group vs. 7.10 ± 0.40 in normoxia group; *p* = 0.44) and physical conditioning sessions (6.05 ± 0.64 in hypoxia group vs. 6.40 ± 0.83 in normoxia group; *p* = 0.30).

### Statistical Analyses

All analyses were performed in R (version 4.0.2) (R Core Team, 2020). For each outcome, a linear mixed-effects model with *time* (Post-0, Post-1, and Post-2), *altitude* (hypoxia group and normoxia group), and their interaction was created, and baseline scores were included as a covariate of no interest (Bates et al., 2015); varied intercepts were permitted by treating subject as a random effect. This model was built for the kinematic variables of the *ippon-seoi-nage* (times and accelerations) and leg extension mechanical variables (peak velocity, jump height, 1RM, maximal theoretical velocity, maximal theoretical load, and load-velocity relationship slope). Since we were principally interested in the between-group average treatment effects of the intervention (altitude), we calculated a contrast for each time point based on the estimated marginal means from the linear mixed-effects model (Lenth, 2020). Residuals were qualitatively examined for heteroscedasticity. Max_accelT was found to have residuals that scaled with fitted values (_ŷ_), so it was log-transformed for analysis and the condition effect was reported in its exponentiated form. This exponentiated effect can be interpreted multiplicatively; hypoxia will have a post-intervention score *x*-times greater than normoxia after adjusting for baseline values. We calculated 90% compatibility intervals (CIs) of the adjusted effects using the bias-corrected and accelerated bootstrap with 500 replicates, resampled on the subject level (Davison and Hinkley, 1997; Efron, 2012; Canty and Ripley, 2019). Magnitude-based standardized mean differences (SMDs) were calculated to complement the inferential statistics. For effects calculated on the raw scale, SMDs were obtained by dividing the between-group adjusted effect by the pooled baseline SDs. For effects that were log-transformed, effect sizes were calculated similarly but on the log (additive) scale. Effect sizes were interpreted using the following classification, as per Hopkins et al. (2009): 0.0, trivial; 0.2, small; 0.6, moderate; 1.2, large; 2.0, very large; and 4.0, nearly perfect.

*p*-Values were calculated using Satterthwaite degrees of freedom.

Similar to previous work (Schoenfeld et al., 2020), we used an estimation-based approach to drawing inferences from our data. Specifically, rather than relying on null hypothesis significance testing and drawing binary conclusions as to the presence of an effect or no effect (Amrhein et al., 2019), we interpreted each effect and its precision continuously (Gardner and Altman, 1986).

All other data that were not used to answer our research questions are presented using descriptive statistics (namely, mean ± SD and geometric mean ⋇ geometric SD).

## Results

**FIGURE 3:**
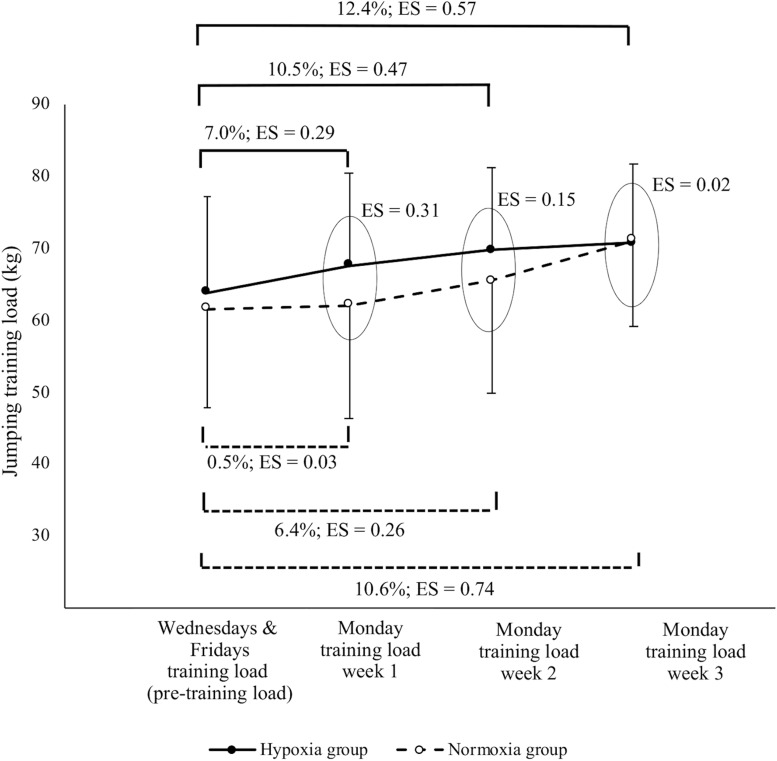
Training load linked to 1.2 m⋅s^–1^ of the mean propulsive velocity throughout the training period. Wednesdays and Fridays’ training load corresponded to the load associated to a mean propulsive velocity of 1.2 m⋅s^–1^ calculated in the pre-training in normoxia; each Monday the training load was adjusted to a mean propulsive velocity of 1.2 m⋅s^–1^ at the corresponding condition; ES, intra and inter-group effect size.

The countermovement jump training load during the study is displayed in Figure 3. Similar moderate improvements in the countermovement jump training load were achieved at the end of the training camp in both groups (adjusted between-group effect of 0.28 kg; CI: −3.72, 3.34). Trivial to small differences in the load linked to 1.2 m⋅s^–1^ of the mean propulsive velocity were found between the hypoxia group and the normoxia group in each of the 3 weeks (ES: 0.31, 0.15, 0.02, respectively). However, the adjusted effect assessed for the training load between groups in the first training session was 4.13 kg (CI: 0.84, 7.29), resulting in a higher value for the hypoxia group (hypoxia group: 7.0%; normoxia group: 0.5%; *p* = 0.03) (Figure 3).

**FIGURE 4:**
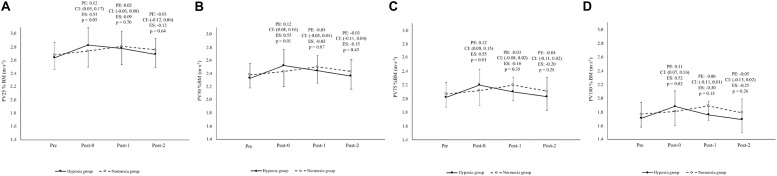
Peak velocities comparison through the study in both groups. PV25%BM **(A)**, PV50%BM **(B)**, PV75%BM **(C)**, PV100%BM **(D)**: peak velocity displacing an overload of 25, 50, 75, or 100% of the body mass. Pre, pre-training; Post-0, post-training; Post-1, 1 week after training; Post-2, 2 weeks after training. PE, adjusted between-group difference [i.e., the estimated marginal mean of the difference between hypoxia and normoxia groups (hypoxia group – normoxia group) at time Post-0, Post-1, or Post-2 after adjusting for baseline differences]; ES, effect size between hypoxia group and normoxia group; *p*-value of the adjusted between-group difference.

Figure 4 shows peak velocity values corresponding to 25–100% of body mass at Pre, Post-0, Post-1, and Post-2, as well as between-group adjusted effects and their respective CIs. The adjusted between-group effects for the peak velocity at the four different loading conditions favored hypoxia group at Post-0 (from 0.11 to 0.12 m⋅s^–1^; CIs from 0.05 to 0.17 m⋅s^–1^). At Post-1 and Post-2, the adjusted between-group effects ranged from a 0.02 m⋅s^–1^ benefit for the hypoxia group to a 0.06 m⋅s^–1^ benefit for the normoxia group, and the CIs around these estimates did not favor either group (ranging from −0.13 to 0.04 m⋅s^–1^). Compared to pre-training, hypoxia group displayed the best performance in peak velocity earlier, at Post-0 (8.8 ± 1.3%), while normoxia group reached this value at Post-1 (5.6 ± 0.7%).

Table 1 shows within-group descriptive statistics for jump height, 1RM relative to body mass and the variables of the load-velocity relationship at the four time points, in addition to between-group adjusted effects and their respective CIs. The adjusted effects for the jump height were high (between 1.81 and 3.22 cm) at Post-0, Post-1, and Post-2 and their respective CIs favored the hypoxia group (ranging from 0.14 to 4.74 cm). Compared to pre-training, hypoxia group displayed the best performance in jump height at Post-0 (8.2 ± 6.9%), while normoxia group reached this value at Post-1 (1.4 ± 5.9%). The maximal theoretical velocity showed an adjusted effect at Post-0 that favored hypoxia group (0.17 m⋅s^–1^; CI from 0.07 to 0.29 m⋅s^–1^). The point estimate of the adjusted effects for 1RM relative to body mass favored the normoxia group mainly at Post-1 and Post-2 (0.15 and 0.20 kg⋅kg^–1^), with CI estimates ranging from 0.03 to a 0.34 kg⋅kg^–1^.

**TABLE 1:** Altitude training effect on maximal dynamic strength, jump height, and load-velocity profile.

		Pre	Post-0			Post-1			Post-2		
	Group	Mean ± SD	Mean ± SD	Adjusted between-group difference (90% CI)	ES (*p*-value)	Mean ± SD	Adjusted between-group difference (90% CI)	ES (*p*-value)	Mean ± SD	Adjusted between-group difference (90% CI)	ES (*p*-value)
1RM (kg⋅ kg^–1^)	Hypoxia	1.86 ± 0.31	1.83 ± 0.30	−0.08 (−0.16, 0.03)	−0.28 (0.34)	1.75 ± 0.29	−0.15 (−0.27, −0.03)	−0.52 (0.08)	1.70 ± 0.35	−0.20 (−0.34, −0.08)	−0.72 (0.03)
	Normoxia	1.87 ± 0.24	1.90 ± 0.21			1.90 ± 0.17			1.85 ± 0.22		
V_0_ (m⋅ s^–1^)	Hypoxia	2.95 ± 0.24	3.20 ± 0.24	0.17 (0.07, 0.29)	0.71 (0.03)	3.12 ± 0.29	0.04 (−0.06, 0.13)	0.18 (0.56)	3.03 ± 0.25	−0.02 (−0.14, 0.09)	−0.06 (0.85)
	Normoxia	2.99 ± 0.22	3.04 ± 0.26			3.12 ± 0.30			3.08 ± 0.28		
L_0_ (kg)	Hypoxia	207.5 ± 30.1	213.8 ± 20.2	4.0 (−11.3, 17.0)	0.13 (0.72)	203.0 ± 37.9	−9.3 (−27.7, 9.8)	−0.31 (0.39)	197.9 ± 25.6	−1.43 (−20.5, 9.3)	−0.05 (0.91)
	Normoxia	195.6 ± 30.9	201.8 ± 37.7			203.8 ± 48.2			194.1 ± 25.6		
Slope (kg⋅ s⋅ m^–1^)	Hypoxia	−71.1 ± 14.7	−67.3 ± 9.7	2.8 (−4.3, 11.1)	0.20 (0.58)	−66.4 ± 19.4	4.2 (−4.9, 13.1)	0.30 (0.41)	−65.3 ± 10.6	0.6 (−5.2, 9.9)	0.04 (0.92)
	Normoxia	−66.0 ± 13.8	−67.1 ± 16.8			−66.7 ± 22.5			−63.5 ± 10.6		
JH (cm)	Hypoxia	34.94 ± 5.02	37.78 ± 5.68	3.22 (1.74, 4.74)	0.59 (0.006)	37.12 ± 5.84	1.81 (0.38, 3.17)	0.33 (0.10)	36.93 ± 5.66	2.01 (0.14, 4.15)	0.37 (0.10)
	Normoxia	37.72 ± 5.97	37.68 ± 5.55			38.21 ± 6.31			37.05 ± 7.06		

*1RM, maximal 1 repetition relative to body mass; V_0_, maximal theoretical velocity; L_0_, maximal theoretical load; Slope, load-velocity relationship slope; JH, jump height; Pre, pre-training; Post-0, post-training; Post-1, 1 week after training; Post-2. 2 weeks after training; adjusted between-group difference is the estimated marginal mean of the difference between hypoxia and normoxia groups (hypoxia group – normoxia group) at time Post-0, Post-1, or Post-2 after adjusting for baseline differences; ES, effect size between hypoxia and normoxia groups; p-value of the adjusted between-group difference.*

Table 2 shows within-group descriptive statistics for times and maximal accelerations reached during the technical performance of the *ippon-seoi-nage* at the four time points, in addition to between-group adjusted effects and their respective CIs. The adjusted effects for the *ippon-seoi-nage*’ times indicate higher times to perform the technique (i.e., a detrimental effect) in hypoxia group compared to normoxia group at all time points assessed (ranging between 56 and 174 ms). The adjusted between-group effect for the acceleration in the phase linked to the leg extension (Max2_accelT) favored normoxia group at all time points (multiplicative adjusted effects in the hypoxia group ranged from 0.73 to 0.91-times the G displayed in the normoxia group).

**TABLE 2:** Altitude training effect on *ippon-seoi-nage* kinematic variables.

		Pre	Post-0			Post-1			Post-2		
	Group	Mean ± SD	Mean ± SD	Adjusted between-group difference (90% CI)	ES (*p*-value)	Mean ± SD	Adjusted between-group difference (90% CI)	ES (*p*-value)	Mean ± SD	Adjusted between-group difference (90% CI)	ES (*p*-value)
Tpeak1_accelT (ms)	Hypoxia	399 ± 69	414 ± 68	71 (32, 114)	1.04 (0.07)	406 ± 108	56 (−3, 119)	0.81 (0.13)	416 ± 93	92 (21, 162)	1.34 (0.04)
	Normoxia	378 ± 67	344 ± 51			351 ± 93			327 ± 88		
Tpeak2_accelT (ms)	Hypoxia	898 ± 126	947 ± 128	132 (62, 196)	1.15 (0.03)	909 ± 132	115 (43, 219)	1.00 (0.05)	940 ± 129	142 (59, 227)	1.24 (0.03)
	Normoxia	809 ± 98	800 ± 74			780 ± 155			790 ± 112		
Tpeak3_accelT (ms)	Hypoxia	1172 ± 102	1192 ± 88	113 (62, 178)	1.04 (0.03)	1168 ± 117	134 (63, 214)	1.23 (0.01)	1242 ± 116	159 (74, 269)	1.46 (0.01)
	Normoxia	1090 ± 115	1066 ± 87			1017 ± 125			1057 ± 124		
Max1_accelT (G)	Hypoxia	6.4 ⋇ 1.25	5.6 ⋇ 1.32	0.96 (0.80, 1.13)*	−0.18 (0.71)	6.2 ⋇ 1.28	1.06 (0.92, 1.25)*	0.26 (0.57)	5.8 ⋇ 1.21	1.08 (0.90, 1.27)*	0.34 (0.51)
	Normoxia	5.6 ⋇ 1.28	5.7 ⋇ 1.27			5.7 ⋇ 1.33			5.5 ⋇ 1.29		
Max2_accelT (G)	Hypoxia	3.0 ⋇ 1.31	2.9 ⋇ 1.42	0.91 (0.72, 1.13)*	−0.35 (0.43)	2.7 ⋇ 1.37	0.73 (0.63, 0.85)*	−1.11 (0.01)	2.4 ⋇ 1.33	0.76 (0.63, 0.90)*	−0.96 (0.05)
	Normoxia	3.6 ⋇ 1.35	3.4 ⋇ 1.41			4.0 ⋇ 1.24			3.8 ⋇ 1.25		
Max3_accelT (G)	Hypoxia	26.4 ⋇ 1.62	29.9 ⋇ 1.29	1.45 (1.19, 1.70)*	0.85 (0.04)	30.4 ⋇ 1.52	1.21 (0.99, 1.58)*	0.45 (0.25)	30.1 ⋇ 1.79	0.99 (0.72, 1.57)*	−0.03 (0.94)
	Normoxia	21.7 ⋇ 1.45	20.2 ⋇ 1.29			23.6 ⋇ 1.46			26.5 ⋇ 1.55		
Thor (ms)	Hypoxia	818 ± 105	898 ± 125	112 (49, 187)	0.87 (0.07)	861 ± 179	113 (29, 203)	0.88 (0.06)	933 ± 174	174 (85, 285)	1.35 (0.01)
	Normoxia	758 ± 152	769 ± 105			729 ± 120			732 ± 96		

Tpeak1_accelT, time from the beginning of the movement to peak 1 of resultant acceleration (AccelT); Tpeak2_accelT, time from the beginning of the movement to peak 2 of AccelT; Tpeak3_accelT, time from the beginning of the movement to peak 3 of AccelT; Max1_accelT, maximal acceleration reached in peak 1; Max2_accelT, maximal acceleration reached in peak 2; Max3_accelT, maximal acceleration reached in peak 3; Thor, time from the beginning of the movement to the horizontal position of the dummy; Pre, pre-training; Post-0, post-training; Post-1, 1 week after training; Post-2, 2 weeks after training; adjusted between-group difference is the estimated marginal mean of the difference between hypoxia and normoxia groups (hypoxia group – normoxia group) at time Post-0, Post-1, or Post-2 after adjusting for baseline differences; ES, effect size between hypoxia and normoxia groups; *p*-value of the adjusted between-group difference. *Indicates multiplicative effects, derived from an exponentiated group effect estimated using a linear model log-transformed data. ⋇, times or divided by, where the value to the left is the geometric mean and the value to the right is the geometric SD; thus, the spread of the data is multiplicative rather than additive (as is the case with ± and a traditional SD). The spread (±1SD) of data that are reported geometrically can thus be calculated as geometric mean ÷ geometric SD and geometric mean × geometric SD.

## Discussion

The aim of this study was to analyze the influence of a power-oriented resistance training program performed at moderate altitude on leg extension capacity and associated technical performance (*ippon-seoi-nage*) in elite judokas. The 3-week training period improved the peak velocity, maximal theoretical velocity and jump height of the countermovement jump both at moderate altitude and at sea level. The hypoxia group produced notable additional improvements in peak velocity, maximal theoretical velocity and jump height after the training period (Post-0), and the additional benefits in jump height were also observed 1 and 2 weeks after training (Post-1 and Post-2). Peak performance in peak velocity and jump height was higher and achieved earlier in hypoxia group (at Post-0) compared to normoxia group (at Post-1). These results suggest that a power-oriented resistance training program performed at moderate altitude seems to accelerate and improve the gains in explosive leg extension capacity. The early achievement of optimum performance in hypoxia group also favors an early loss of some of the neuromuscular adaptations obtained, which was particularly evident in the ability to produce maximal force as displayed in the 1RM values at Post-1 and Post-2. Concerning the kinematic analysis of the *ippon-seoi-nage*, the hypoxia group elicited an increased time of execution and reduction in acceleration in the test when compared to normoxia group, indicating a detrimental effect of altitude training on a highly coordinated technique. These results suggest that although power-oriented resistance training at moderate altitude accelerates and improves explosive leg extension capacity, the altitude itself and/or the derived physiological changes should be taken into account in program design for skill acquisition (i.e., technique training) during altitude training in sports with complex coordination movements.

Resistance training under conditions of hypoxia is in an early stage of research and the available studies on this topic are scarce and varied making it difficult to draw strong conclusions. Some natural altitude resistance training studies did not include a control group at sea level (García-Ramos et al., 2014, 2016a), used other types of training, such as concurrent training (García-Ramos et al., 2016b), or applied power resistance training but under an intermittent strategy (Morales-Artacho et al., 2018). In this regard, a 3-week concurrent training at moderate altitude did not produce changes in the peak velocity of the squat jump of elite swimmers (García-Ramos et al., 2016b). Two weeks of leg power resistance training at moderate altitude induced an improvement of 4.4% in the peak velocity of the squat jump (García-Ramos et al., 2014) and of 7.2% in squat jump height (García-Ramos et al., 2016a) in elite swimmers. Similarly, our elite judokas achieved higher increases in peak velocity at different percentages of body mass and in countermovement jump height after 3 weeks of power resistance training in hypoxia group than in normoxia group (mean difference between conditions of 3.2% for peak velocity and 6.8% for countermovement jump height). Increasing terrestrial altitude reduces air density (∼3% for every 350 m) and consequently the air resistance (Levine et al., 2008), which benefits the performance of explosive actions (Hollings et al., 2012). Thus, improvements in explosive actions at moderate altitude have been previously reported in basic strength exercises (bench-press, squat, squat jump, and countermovement jump) (Chirosa et al., 2006; Feriche et al., 2014; García-Ramos et al., 2018) and in high-velocity sporting activities (sprints, jumps, and throws) (Levine et al., 2008; Hollings et al., 2012; Hamlin et al., 2015). Considering that air resistance is approximately proportional to the square of the speed (Frohlich, 1985), athletes competing in events with faster running speeds (e.g., 100 m) are expected to benefit more from the reduced air density associated with altitude (Hamlin et al., 2015; Girard et al., 2017). Therefore, in this study, although at lower magnitude (the peak velocity reached during the actions evaluated was ∼2–3 m⋅s^–1^), an altitude-induced benefit was observed on explosive leg extension from the first week of exposure. The combined effect of the reduction in air density and the neuromuscular effects related to altitude ascent (Melissa et al., 1997; Schoenfeld, 2013; Scott et al., 2014b) potentially explain the differences between the results of the present study and those achieved at normobaric simulated hypoxia, where no added benefit in power/strength performance has been reported (Feriche et al., 2014; Scott et al., 2015).

Acute exposure to moderate altitude did not produce changes in the kinematic variables of the *ippon-seoi-nage*, although analysis of the coefficient of variation ratio suggested a change in the space-time pattern of the technique (Almeida et al., 2018). In the present study, 3 weeks of an altitude training camp actually had a negative effect on *ippon-seoi-nage* technical performance. Thus, compared to sea-level training, judokas showed impaired times of execution of the technique, as well as a lower acceleration during the phase linked to the leg extension (Max2_accelT). These results are contrary to what we expected, since other studies (García-Ramos et al., 2016a,b) reported technique performance improvements in swimmers after altitude training. Disparities between findings are likely due to differences in the complexity of the movement, considering that the *ippon-seoi-nage* is a highly complex technique (Almeida et al., 2018; Ishii et al., 2018). Accordingly, the swimming start can be classified as a simple technical maneuver, especially in elite swimmers, and thus the adaptations achieved from training at moderate altitude would conceivably have greater transfer to improved performance (García-Ramos et al., 2014, 2016a,b). Moreover, the swimmers spent 13.4% of their pool session time in specific starting technique training (García-Ramos et al., 2016a), whereas in this study judokas only spent 10.9% of the resistance training session time in specific technique training (18–24 *ippon-seoi-nage* throws 3 times per week). Motor learning strategies are required to transfer improvements in strength to skilled performance (Suchomel et al., 2016). The length of time needed to achieve this transfer or lag time seems to be higher after altitude training. This is conceivably due to a higher change percentage observed in the explosive leg extension capacity in hypoxia group, requiring more time to accommodate bigger changes, and also because altitude leads to changes in the space-time pattern of the technique that require time to adjust (Almeida et al., 2018). For this reason, transference work with specific technique exercises to allow the adjustments in its space-time pattern and to integrate the changes in physical performance into the technical performance should be included in the training program at this environmental condition.

There were no notable changes in the 1RM (kg.kg^–1^ of body mass), maximal theoretical load and load-velocity slope just after the training program or between groups. This result was expected since this mesocycle was designed to enhance explosive muscle performance (Cormie et al., 2011) rather than maximal strength. Notwithstanding, including moderate to high-load exercises (70–90% 1RM) after the main part of the resistance training sessions allowed participants to maintain strength levels over the course of the intervention. Using the countermovement jump pre-training load linked to 1.2 m⋅s^–1^ of the mean propulsive velocity allowed us to orientate the resistance training program toward velocity enhancement on two of the three weekly training days. Indeed, large improvements in jump capacity were observed in both training conditions but clearly more accentuated in hypoxia group compared to normoxia group. Thus, large increases in peak velocity were observed in both groups, displaying a mean increase in performance from pre-training to Post-0 of 8.8% in hypoxia group (ES: 0.78) and 5.6% to Post-1 in normoxia group (ES: 0.57). Moreover, having the training load readjusted to maintain the mean propulsive velocity of 1.2 m⋅s^–1^ at the corresponding condition once a week induced a clear tendency to increase this load with respect to the pre-training in hypoxia group from the first week (hypoxia group: 7.0%; normoxia group: 0.5%), while the normoxia group only showed appreciable changes from the second week. This type of response in the absolute training load has been previously observed, highlighting the need to adjust the training load during maximal-velocity resistance training at different altitude levels (>1500 m) (Feriche et al., 2017; Morales-Artacho et al., 2018; Rodríguez-Zamora et al., 2019).

The characteristics of the training program used in this study and the altitude effect likely explain why the peak velocity and jump height increased and why the magnitude of the improvements in the hypoxia group was higher and achieved earlier than in normoxia group. It is possible that altitude training accelerates neuromuscular adaptations, due to a rise in the recruitment of type II fibers (Melissa et al., 1997; Schoenfeld, 2013; Scott et al., 2014b) or an increase in spinal excitability (Delliaux and Jammes, 2006; Amann et al., 2013; Tomazin et al., 2016). Similarly, Nishimura et al. (2010) found that resistance training under normobaric hypoxia improves muscle strength quicker than under normoxia (3 vs. 6 weeks). According to Millet et al. (2010), a variability in aerobic performance is observed by coaches after the return to sea level. Aerobic peak performance is reportedly seen in some athletes from the 2nd to 4th day after returning to sea level, followed by a decrease up to the 15th–21st day (Chapman et al., 2014). Although there is a paucity of research on the post-altitude muscle power behavior, the best results in our study were reached the day participants returned to sea level (Post-0). Two weeks after the return to sea level (Post-2), the results from both groups tended to return to pre-training values with no substantial differences between them despite the better and earlier peak performance achieved in hypoxia group. The loss of the neuromuscular adaptations also started earlier in hypoxia group, particularly in the application of maximum level of strength once training with heavy loads was abandoned (Cormie et al., 2007). Further research is warranted to explore potential differences in the dynamics of training-induced responses and to clarify the principal mechanisms involved in muscle power-related training adaptations.

A potential limitation of this study includes possible confounding from a placebo effect. However, it should be noted that the judokas were not aware or given any information about the effects of altitude exposure on performance. Another limitation was that the study did not comprise a mid-intervention assessment and thus it was not possible to determine if peak performance could be achieved before Post-0.

## Data Availability Statement

The raw data supporting the conclusions of this article will be made available by the authors, without undue reservation.

## Ethics Statement

The studies involving human participants were reviewed and approved by the Research Ethics Committee of the University of Granada. The patients/participants provided their written informed consent to participate in this study. Written informed consent was obtained from the individual(s) for the publication of any potentially identifiable images or data included in this article.

## Author Contributions

PP, JB-G, and BF contributed to the conception and design of the study. All authors participated in data base collection. FA and BF organized the database and performed the statistical analysis. FA wrote the first draft of the manuscript. PP and BF supervised the study. All authors contributed to manuscript revision, read and approved the submitted version.

## Conflict of Interest

The authors declare that the research was conducted in the absence of any commercial or financial relationships that could be construed as a potential conflict of interest.
